# Psychophysiological, but Not Behavioral, Indicator of Working Memory Capacity Predicts Video Game Proficiency

**DOI:** 10.3389/fnhum.2021.763821

**Published:** 2021-10-28

**Authors:** Natalia Jakubowska, Paweł Dobrowolski, Alicja Anna Binkowska, Ibrahim V. Arslan, Monika Myśliwiec, Aneta Brzezicka

**Affiliations:** ^1^Department of Psychology, SWPS University of Social Sciences and Humanities, Warsaw, Poland; ^2^Polish-Japanese Academy of Information Technology, Warsaw, Poland; ^3^Institute of Psychology, Polish Academy of Sciences, Warsaw, Poland

**Keywords:** action video games, visual working memory, trainings, ERPs, EEG

## Abstract

Visual working memory (VWM) is the ability to actively maintain visual information over short periods of time and is strongly related to global fluid intelligence and overall cognitive ability. In our study, we used two indices of visual working memory capacity: the behavioral estimate of capacity (K) and contralateral delay activity (CDA) in order to check whether training in a Real-Time Strategy (RTS) video game StarCraft II can influence the VWM capacity measured by the change detection task. We also asked a question whether individual differences in behavioral and psychophysiological indices of VWM can predict the effectiveness of video game training. Sixty-two participants (non-players) were recruited to the experiment. Participants were randomly assigned to either experimental (Variable environment), active control (Fixed environment), and passive control groups. Experimental and active control groups differed in the type of training received. Training consisted of 30 h of playing the StarCraft II game. Participants took part in two EEG sessions (pre- and post-training) during which they performed the VWM task. Our results showed that working memory capacity (K calculated according to Pashler’s formula) increases after training in both experimental groups, but not in a control group. We have also found a correlation between average visual working memory capacity (calculated as K) and mean CDA amplitude no matter which group we are looking at. And, last but not least, we have found that we can predict the amount of improvement in the RTS video game by looking at the psychophysiological indices (CDA amplitude) recorded at baseline (before training), but only in the experimental group. We think that the strength of the psychophysiological indicator of VWM capacity might be a marker of the future success in video game acquisition.

## Introduction

Visual working memory (VWM) allows us to maintain visual information over short periods of time for manipulation or later access (Baddeley, 2003; D’Esposito and Postle, 2015). VWM is an important cognitive function in our daily life and is essential for many higher-level cognitive processes, like problem-solving, learning by observation, or reading (Fukuda et al., 2010; Shipstead et al., 2012). The capacity of VWM relates to the amount of visual information, which can be maintained in memory simultaneously and accessible if needed (Luck and Vogel, 2013). Previous research (including neuroimaging studies) has shown that VWM capacity is highly limited (Luck and Vogel, 1997; Todd and Marois, 2004), differs across individuals (Rouder et al., 2008), and predicts fluid intelligence in adults (Fukuda et al., 2010; Unsworth et al., 2014). Studies on VWM have relied on a well-established paradigm that measures VWM capacity—the *change detection task* (Luck and Vogel, 1997, 2013), where participant maintains a visual image in memory over a short delay interval and answers if any item (or items) in a later probe image have changed compared to the sample image. The number of items presented (memory load) is manipulated, and performance (working memory capacity, an estimate of the number of items stored in WM measured by K calculated according to Pashler’s formula in our study) is compared between trials of different loads. Change detection accuracy mirrors a participant’s limitation of VWM capacity and is usually limited to 3–4 items (Vogel and Awh, 2008). It is suggested that the limitation of VWM capacity is associated with visual search and multiple-object tracking performance (Drew et al., 2011; Luria and Vogel, 2011). Previous research has shown that participants with higher VWM capacity are more effective in ignoring unnecessary items during task performance (Vogel et al., 2005). In neurophysiological studies of lateralized VWM, stimuli are presented peripherally, and the subject’s task is to attend and maintain in VWM only the items presented in a cued visual hemifield. This generates a lateralized representation, which is larger contralateral compared to ipsilateral of the memorized hemifield, in posterior cortical areas over the retention period that results in a contralateral delay activity (CDA). CDA is a negative slow-wave evoked component that amplitude relates to the number of objects maintained in VWM, so it could be interpreted as a neural index of WM load (Vogel and Machizawa, 2004; Luria et al., 2016). Previous research has shown that CDA amplitude is correlated with memory capacity (Vogel and Machizawa, 2004; Ikkai et al., 2010) and can be modified as a result of WM training (Li et al., 2017). In this study, we used video games as a specific kind of cognitive training having the potential for VWM improvement.

The growing body of research suggests that playing video games enhances the performance on tasks measuring visual and attentional abilities (Green and Bavelier, 2007; Jakubowska et al., 2021). Potential cognitive benefits are possible even with relatively short periods of engagement in playing activity (Green and Bavelier, 2007; Wilms et al., 2013), which makes video games an attractive training option for restoring cognitive functions following brain impairments and in preventive cognitive interventions (Achtman et al., 2008). As there are different kinds of video games, the particular category called action video gaming (AVG) is thought to have a substantial impact on human cognitive functioning. AVG requires players to scan many different complex visual stimuli at the same time and react to multiple stimuli or situations under time pressure (Green and Bavelier, 2003, 2012). AVG is cognitively demanding because of engaging many cognitive functions like working memory, visual attention, and inhibitory control (Green and Bavelier, 2003, 2012). Previous research has shown that long experience in AVG was associated with VWM improvement measured with a change detection task (Boot et al., 2008; Blacker et al., 2014; Li et al., 2015) as well as other tasks (Colzato et al., 2010; Sungur and Boduroglu, 2012; Waris et al., 2019). These results suggest that AVG training may lead to the enhancement of VWM. At the same time, VWM is a key cognitive function in effective video gaming, because it allows players to keep task-relevant visual stimuli over short periods of time for manipulation or later access (Logie, 2011; Blacker et al., 2014). Noteworthy, some studies suggest that cognitive enhancement connected to video game playing does not show far transfer’s characteristics (like general improvement in cognitive functioning or learning), but seems to be limited to functions being involved in a given type of video game (Oei and Patterson, 2014).

It is important to note that there are studies that have not found a cognitive improvement after gaming training (Seçer and Satyen, 2014; Dominiak and Wiemeyer, 2016). The possible explanation of these divergent results could be connected to different kinds of games being considered as AVG is actually a broad category with wide inclusion criteria. The study conducted by Dobrowolski et al. (2015) has shown that the achievement of expertise in two different game genres, while both included in AVG category called real-time strategy (RTS) and first-person shooter (FPS), impacts differently cognitive functioning of players. The higher performance in task engaging visual attention and task-switching ability were observed only in RTS (but not in FPS) players as compared to non-players (Dobrowolski et al., 2015). Similarly, RTS experts seem to have higher accuracy and larger VWM capacity than non-experts (Yao et al., 2020). The possible interpretation of these results is that video gaming-related cognitive benefits may depend on the type of actions performed within the game (Dobrowolski et al., 2015). As RTS gaming requires extensive interaction with the complex visual environments, we assume it is highly possible to improve VWM through training with this type of video game. While previous investigations indicate that AVG experts have larger visual attentional capacities, greater capacity of working memory, and higher visual acuity as compared to non-gamers (Green and Bavelier, 2003, 2012; Oei and Patterson, 2013), and that specific AVG can positively affect the level of a given function (Bejjanki et al., 2014; Choi et al., 2020), the impact of the initial level of cognitive functioning on player performance remains largely unexplored.

That is why we decided to use the (RTS) video game StarCraft II with two different types of environments requiring diverse cognitive workloads. Our training types were based on either variable or fixed game environments. The opponent’s faction and strategy varied in the variable environment group only (and it was connected to the higher level of difficulty). Our participants were randomly assigned to either a Variable environment, a Fixed environment or the control group. Then it’s important to mention that differences between variable and fixed training models were investigated in previous studies, which proved that variable training enhances learning rates and retention, and induce transfer to untrained tasks more, effectively than fixed training (Kramer et al., 1999; Bherer et al., 2008; Erickson et al., 2010). Moreover, training based on a variable environment seems to have more in common—than training with a fixed environment—with people’s gaming experiences in everyday life.

The objective of the current study was to investigate the impact of RTS video game StarCraft II training on VWM capacity by comparing training groups’ and control group’s behavioral (k estimate of WM capacity) and ERP (contralateral delay activity) data in a change detection task. Furthermore we were also interested in whether initial, individual differences in behavioral and psychophysiological indices of VWM can predict the effectiveness of video game training, which could extend our knowledge of the relationship between VWM and in-game performance.

## Materials and Methods

### Participants

A total of 104 participants were recruited online *via* a covert questionnaire (Sobczyk et al., 2015). As a result of: (1) resignation (*n* = 13); (2) wrong hardware configuration (*n* = 7); (3) failure to meet all training objectives (*n* = 6); (4) bad quality of data (*n* = 7); and (5) lost data (*n* = 9) only 62 of participants were included in analyses reported here. Participants were randomly assigned to two training groups: with Variable Environment training (VEG; *n* = 22; 12 males; *M*_age_ = 25.05, *SD*_age_ = 2.97), with Fixed Environment training model (FEG; *n* = 21; 8 males; *M*_age_ = 25.33, *SD*_age_ = 3.01), and to two control groups: passive control (PC) group (*n* = 8; 5 males, *M*_age_ = 24.63, *SD*_age_ = 2.97), that did not receive any training and active control (AC) group (*n* = 11; males = 8; *M*_age_ = 25.55, *SD*_age_ = 4.41). The participants played Heart Stone for 30 h (8 h in the laboratory and 22 h at home). As the size of the control groups was inappropriate to analyze them individually, and neither 4 × 2 × 2 repeated measures ANOVAs with Load and Session as the within-subjects factors and Group as the between subject factor, nor One-way ANOVAs with Group as a factor showed any between group differences on behavioral or neurophysiological levels, we decided to merge the groups into one Control group (CG; *n* = 19; 13 males; *M*_age_ = 25.16, *SD*_age_ = 3.80). Then it is important to mention that dropout, which largely contributed to the reduction of the size of the control groups, is a common problem in longitudinal training studies (e.g., Moore et al., 2017). Furthermore, our study employed restrictive recruitment criteria, especially in terms of experience in video game playing, which finally resulted in an inability to re-complete the control groups. All participants reported normal or corrected-to-normal visual acuity, normal color vision and normal hearing. They were right-handed and reported not being on any medications, no history of neurological or psychiatric disorders and injuries, including no previous head trauma, no previous head or neck surgery, and no brain tumors. All participants declared less than 5 h of video games played per week over the past 6 months and no experience with Real Time Strategy or First Person Shooter games. Informed consent was obtained from each participant before the start of the experimental procedure.

### Procedure

**Figure 1:**
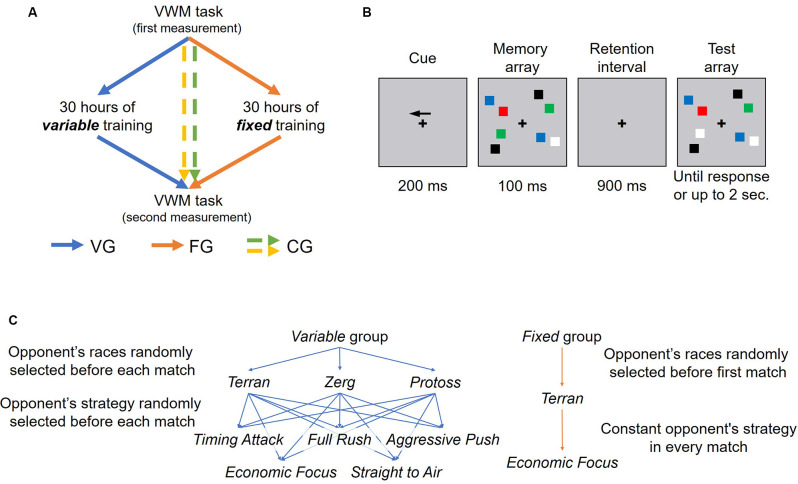
**(A)** Study design: two measurement sessions were carried out during the study (pre-training and post-training). Training included 30 h of playing in the real-time strategy game (*StarCraft II*), spread over 4 weeks. Training varied depending on the group. The Control group (CG) merges participants from Passive Control, who participated only in the measurement sessions and Active Control, who additionally played Heart Stone for 30 h (8 h in the laboratory and 22 h at home). **(B)** The visual-working memory task. Participants directed their attention to a cued hemifield (left of right, guided by an arrow at the beginning of each trial) and compared two arrays of colored squares (memory and test arrays) separated by a retention interval. The test array was either identical to the memory array (no-change condition) or differed by one color (change condition). Participants answered whether the two arrays were identical or not. **(C)** While all of the participants from training groups played as a Terran faction during training, the opponent’s race and strategy varied according to the training group type. Participants from the Variable Environment Group (VEG) could match three factions, from each could use one of five strategies. The faction and the strategy were randomly selected before each match for the Variable group. In the case of a Fixed Environment Group (FEG), participants always played against the Terran faction, which used an economic strategy.

The study design and the informed consent form were approved by the Ethics Committee of the SWPS University of Social Sciences and Humanities. The research consisted of three steps: (1) Pre-training measurement of cognitive function *via* change detection task (Visual Working Memory task; VWM); (2) Training sessions applied to active groups; and (3) Post-training measurement (Figure 1). Experimenters were present during all meetings. Measurement and training sessions took place in the laboratories of the SWPS University in Warsaw.

### Experimental Procedure

Prior to the beginning of the experiment, participants were verbally instructed as to what they would be experiencing and were shown what the procedure of EEG electrode mounting entails. Then, after signing a consent form, participants were brought into a laboratory setting and seated in front of a 24 inch BenQ XL2411Z computer monitor (1,920 × 1,080 resolution, 100 Hz refresh rate) at a distance of 60 cm. Electrodes were then mounted and participants were briefly shown the EEG signal and explained how it is affected by eye blinks and muscular movements, which was a part of the procedure aimed at minimizing the number of artifacts in the signal. The procedure was then started, and upon its completion subjects were provided with a place to wash their hair. The entire procedure lasted no more than 2 h and was identical during both measurements. All subjects, who fulfilled training requirements and participated in both measurement sessions, were compensated for their participation with approx. 184 USD after post-training measurement.

### Experimental Task—Change Detection Task Paradigm

The experimental task was based on the procedure outlined by Vogel and Machizawa (2004). An initial fixation cross was followed by an arrow, pointing which side of the screen needs to be attended (whether right or left hemifield), after which a pattern (memory array) of two to five colored squares appeared in each hemifield of the screen. The same array appeared again (test array) after a brief retention interval, with a 50% chance that one of the squares in the cued hemifield changed its color. Participants were tasked with detecting changes between the memory and test array by responding with one of the keys (same or different). Square colors were chosen at random from seven possibilities (red, blue, violet, green, yellow, black, white), with the constraint that one color appeared no more than twice in a given test array. Squares (0.65 × 0.65 visual degrees) were randomly positioned at the start of each trial in two 4 deg. × 7.4 deg. hemifields (centered 3 deg. to the left and right of a central fixation, light gray background), with a minimum 2 deg. (center to center) distance between squares. All participants completed 576 trials (144 per load) of the task along with 16 initial practice trials.

### Training

#### StarCraft II Training

The StarCraft II (SC2) training consisted of 30 h of training time over a 4-week period. Training consisted of playing matches (approx. 20 min each) against SC2’s artificial intelligence (AI), and all matches were played at our laboratory. Training objectives required the participants to train a minimum of 10 h per week, but no more than 5 h per day. This was done to avoid excessive skew in the distribution of training hours across the training period. There were also two possible training types: Fixed and Variable. The exact differences between the types of training are described below and were presented in Figure 1.

Participants had to access an online platform before each match in order to receive configuration parameters; the parameters consisted of the difficulty setting, the opponent’s faction, the opponent’s strategy, and the game map. Participants from both groups played all of their matches as a Terran faction. While the map was randomly selected from 14 maps before each match in both—Fixed and Variable—training versions, the opponent’s faction, and strategy only varied in the Variable group. The Fixed group always faced the same faction (Terran), and their opponent always applied a more passive “Economic Focus” strategy. The Variable group could face any of the three factions (each with their own unique units and abilities) and also any of five opponent strategies: Full Rush, Timing Attack, Aggressive Push, Economic Focus, Straight to Air. The game difficulty was set adaptively for both training types spanning across eight levels (1. Very Easy; 2. Easy; 3. Medium; 4. Hard; 5. Harder; 6. Very Hard; 7. Elite; 8. Cheater) The online platform software recorded the number of wins (+1) and losses (−1) and each time the total passed the multiple of four threshold, the difficulty was increased by one. The difficulty decreased whenever the total dropped below the multiple of four threshold. The training was preceded by an introduction phase designed to familiarize participants with the core concepts of the game and basic gameplay mechanics (see “StarCraft II Introduction” section).

#### Starcraft II Introduction

The introductory phase consisted of eight parts: (1) a short text describing the goals of the meeting; (2) a text and video-based description of the overall game; (3) a video introduction to the Terran faction, its units and buildings; (4) a text-based description of the fundamental game concepts and in-game interface; (5) an AI guided tutorial that introduces the gameplay in real time, allowing participants to experience the game for the first time; (6) a quiz requiring that the correct labels be attached to each of the five basic unit and building types that are available to the Terran faction, which was intended to check if participants were attentive to the training materials; (7) two films (25 min. each) describing basic strategies and explaining the various stages that each match progresses through; and (8) a three-match series in which the game progressively increased its difficulty, speed, and available units, with no specific guiding instructions. The entire introduction lasted approx. 2.5 h, and did not count into the required 30 h of training. It was also automated and self-paced, with experimenters only providing assistance when needed and also during part 8 of the introduction where assistance was provided to keep up the pace and direction of each training game. Upon completion of this introduction, participants were free to begin training on the following day.

### EEG Recording and Analysis

A 64-channel SynAmps RT Neuroscan EEG amplifier and BrainProducts actiCap Ag/AG-Cl active electrode set were used to record brain activity during task performance. All channels were recorded at 1,000 Hz sampling rate. Impedances were held below 5 kΩ. All data were preprocessed offline using MATLAB environment and EEGlab (Delorme and Makeig, 2004), and ERPlab (Lopez-Calderon and Luck, 2014) software packages. The signal was initially re-referenced to a common average and then down-sampled to 250 Hz, followed by a band-pass filter between 0.1 and 40 Hz. Data epochs between −0.2 and 0.996 s were extracted, and all epochs with incorrect behavioral responses were rejected. The remaining epochs were manually filtered for eye-blinks/movements and excessive muscle activity and then averaged.

### Data Reduction and Analysis

All analyses were conducted using R Statistical Software (Foundation for Statistical Computing, Vienna, Austria), IBM Corp. Released 2017. IBM SPSS Statistics for Windows, Version 25.0. Armonk, NY: IBM Corp, python and MATLAB custom scripts.

Mixed ANOVAs (3 × 4 × 2) were used to analyze the behavioral and neurophysiological data including the between group variables of group (three levels: CG *vs*. FEG *vs*. VEG) and the within group variables of load (four levels: 2 *vs*. 3 *vs*. 4 *vs*. 5) and session (two levels: pre-training measurement *vs*. post-training measurement). Group comparisons for telemetric data were conducted by a series of t-tests (two-group comparisons). *Post hoc* pairwise t-tests were also performed in case of significant main effects or interactions, with Bonferroni correction for multiple comparisons.

Telemetric data were collected from a total of 5,494 games. While SC2 replays allow obtaining dozens of different variables, participants’ expertise or game results do not depend on any particular one. Nevertheless, we selected basic predictor variables that relate to cognitive-motor abilities and game proficiency. We focused on (1) the number of matches played by each player; (2) first army unit creation latency; and (3) first supply collection latency. As better SC2 players play shorter matches, the first of mentioned variables should reflect general players’ proficiency. It should be emphasized that the number of played matches positively correlated with the number of won matches (*r* = 0.964, *p* < 0.001) and matches played on more difficult levels (Harder: *r* = 0.458, *p* = 0.002; Very hard: *r* = 0.634, *p* < 0.001, Elite: *r* = 0.605, *p* < 0.001; Cheater: *r* = 0.595, *p* < 0.001), but not easier ones (Very Easy: *r* = −0.145, *p* = 0.354; Easy: *r* = −0.146; *p* = 0.351; Medium: r −0.239, *p* = 0.122; Hard: *r* = −0.019; *p* = 0.904). Then it can be assumed that a higher number of played matches is due to players’ higher skills rather than multiple lost matches. Latencies of first army unit creation and first supply collection relate to two key moments in the game environment, which faster execution should result in better performance in the game. We also calculated the overall time each player spent in the game environment which allowed us to confirm the fulfillment of training assumptions. All mentioned telemetric variables were tested for between-group differences by a series of t-tests.

For behavioral data, the capacity of visual working memory, which is measured by the K value, was calculated using the formula proposed by Pashler (1988).

where P(hit) = hits/(hits + misses), and P(FA) = false alarms/(false alarms + correct rejections). In addition to the K values of each set size, we also computed the average K value (K_mean_) for each participant’s visual working memory capacity.

For neurophysiological data, mean amplitudes of CDA (lateralized waveforms; contra—ipsi), averaged across P7/P8 electrodes, from 400 to 900 ms time window were outcome variables (Figures 4A–C).

**Figure 2:**
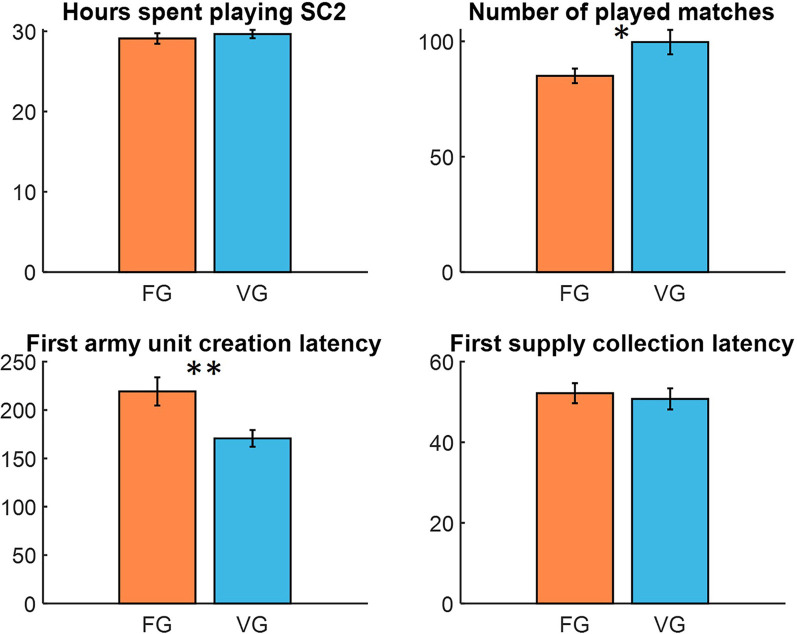
Telemetric variables obtained from the StarCraft II (SC2) environment. While hours spent playing SC2 (upper left) allows us to confirm that both groups (VG: Variable Group; FG: Fixed Group) fulfill training assumptions, other variables indicate players’ proficiency. Barplots presenting first army unit creation (lower left) and first supply collection (lower right) give latencies in seconds. Asterisks indicate statistical significance: *p < 0.05, **p < 0.01.

**Figure 3:**
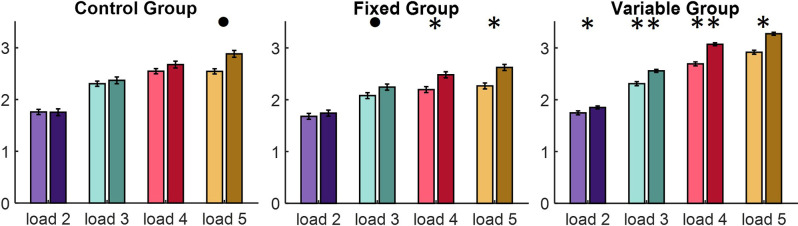
The average K values for each set size in the two tests presented separately for each group. Lighter colors in the pair correspond to the pre-training measurement and darker to the post-training measurement. Asterisks indicate statistical significance: ^•^*p* < 0.08, *p < 0.05, **p < 0.01.

**Figure 4:**
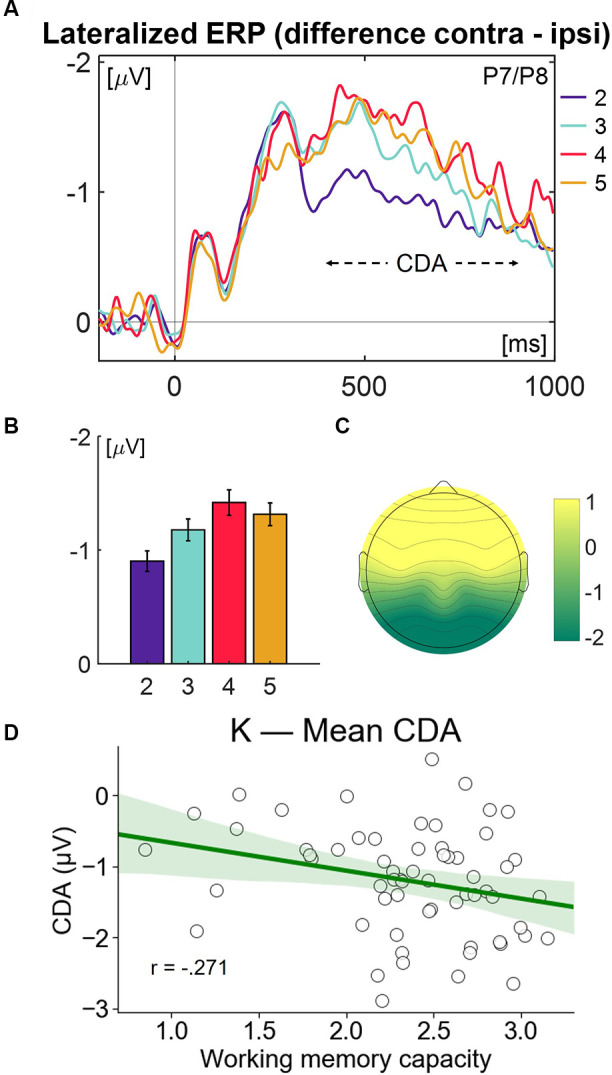
**(A)** Grand average lateralized waveforms (contra—ipsi), averaged across P7/P8 electrodes, separately for all lateralized target distributions. For statistical analyses of CDA, the mean amplitude from 400 to 900 ms was used. **(B)** Mean CDA amplitude from 400 to 900 ms, separately for each load. Error bars denote standard errors of the mean, corrected with within-subjects comparisons. **(C)** Topography of the average activity at each electrode site from 400 to 900 ms. As values were averaged across paired electrodes, the topography is perfectly symmetrical. **(D)** Scatterplot of working memory capacity (K) averaged across loads and contralateral delay activity (CDA) averaged across loads.

To examine the relationship between behavioral, psychophysiological, and telemetric data, linear regression analyses were conducted.

## Results

### Telemetric Data

We started by calculating the total time spent in the game and the mean number of played matches for each player. Although there were no significant difference between groups in time spent playing SC2 (*p* = 0.513), participants from Variable group were able to play significantly more matches in that time period (VEG: Mean = 99.68, *SD* = 24.782; FEG: Mean = 85.05, *SD* = 14.5); *t*_(34.152)_ = 2.376, *p* = 0.023 (Figure 2). Then we calculated the mean latencies of first army unit creation and first supply collection. Analysis revealed that participants from the Variable group created their army units significantly faster (VEG: Mean = 170.685, *SD* = 40.505; FEG: Mean = 219.243, *SD* = 66.683); *t*_(41)_ = −2.901, *p* = 0.006, but there were no differences in the latency of the first supply collection (*p* = 0.696).

### Behavioral Data

The capacity of visual working memory, measured by the K values, were analyzed using a 4 (Load: load 2 *vs*. load 3 *vs*. load 4 *vs*. load 5) × 2 (Sessions: pre-training *vs*. post-training) × 3 (Group: Control *vs*. Fixed *vs*. Variable) repeated-measures ANOVA, with Load and Session as the within-subjects factors and Group as the between subject factor (Figure 3).

Analysis revealed the main effects of Group [*F*_(2, 59)_ = 3.209, *p* = 0.048, *η*^2^ = 0.1], Load [*F*_(3, 57)_ = 134.515, *p* < 0.001, *η*^2^ = 0.49], Session [*F*_(1, 59)_ = 30.22, *p* < 0.001, *η*^2^ = 0.07], Load * Session interaction [*F*_(3, 57)_ = 2.808, *p* = 0.039, *η*^2^ = 0.02] and Group * Load interaction [*F*_(6, 116)_ = 3.992, *p* < 0.001, *η*^2^ = 0.05] but no Load * Session * Group interaction [*F*_(6, 116)_ = 1.806, *p* = 0.104, *η*^2^ = 0.085] or Session * Group interaction [*F*_(2, 59)_ = 0.541, *p* = 0.585, *η*^2^ = 0.018].

Additional analyses revealed that, while Control group wasn’t able to significantly increase its capacity of visual working memory at any of used loads, Fixed group increased it at the load 4 (*p* = 0.025) and load 5 (*p* = 0.049) and Variable group was able to significantly increase it at every load (load 2, *p* = 0.029; load 3, *p* = 0.008; load 4, *p* = 0.003, load 5, *p* = 0.044).

### Psychophysiological Data

Contralateral delay activity was analyzed using a 4 (Load: load 2 vs. load 3 vs. load 4 vs. load 5) × 2 (Sessions: pre-training vs. post-training) × 3 (Group: Control vs. Fixed vs. Variable) repeated-measures ANOVA, with Load and Session as the within-subjects factors and Group as the between subject factor.

Analysis revealed that the only significant effect was the Load effect [*F*_(3, 57)_ = 89, *p* < 0.001, *η*^2^ = 0.288], but no Session [*F*_(1, 59)_ = 0.087, *p* = 0.769, *η*^2^ = 0.002], Group [*F*_(2, 59)_ = 2.212, *p* = 0.118, *η*^2^ = 0.02], Load * Session interaction [*F*_(3, 57)_ = 1.336, *p* = 0.272, *η*^2^ = 0.066], Load * Group interaction [*F*_(6, 116)_ = 0.412, *p* = 0.87, *η*^2^ = 0.021] or Session * Group [*F*_(2, 59)_ = 0.667, *p* = 0.517, *η*^2^ = 0.022].

### Psychophysiological, Telemetric, and Behavioral Data Relations

**Figure 5:**
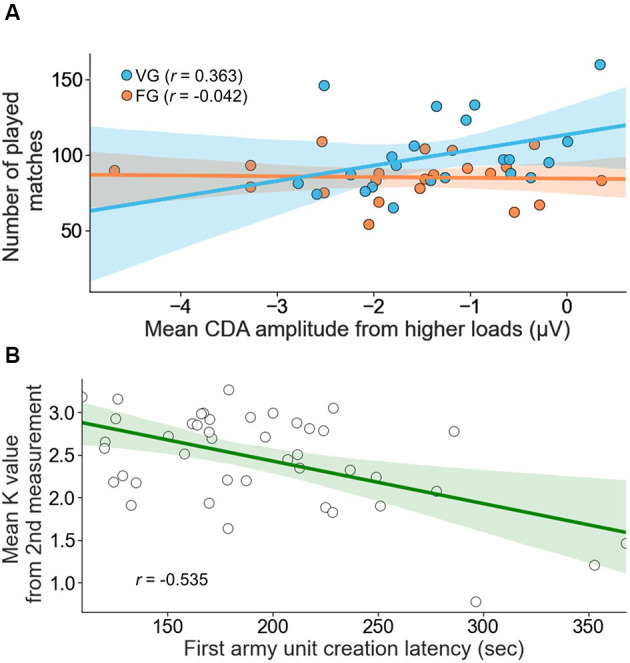
In-game behaviors relationship to psychophysiological and behavioral variables. **(A)** Initial (from the 1st measurement point) contralateral delay activity (CDA) averaged across loads 4 and 5 predicts the overall number of played matches in the Variable Environment Group (VEG). Observed effect turned out to be insignificant in the Fixed Environment Group (FEG) only. **(B)** The averaged across training latency of first army unit creation predicts participants’ mean K value obtained in post-training measurement. The effect was significant in both groups.

In the next step, we created a model containing a mean contralateral delay activity (CDA) averaged across loads 4 and 5 obtained from pre-training measurement as a predictor, Group as a moderator variable and mean number of played matches as a dependent variable. Created model turned out to be significant [*F*_(3, 39)_ = 3.387, *p* = 0.028, *R^2^* = 0.207] and contained significant influence of the Group [*b* = 29.077, *t*_(39)_ = 2.68, *p* = 0.011] and tendency of interaction between CDA and Group [*b* = 10.736, *t*_(39)_ = 1.734, *p* = 0.079]. Next, it was revealed that while there was no relationship between CDA and number of played matches in the Fixed Group (*p* = 0.891), there was a significant negative relationship in the Variable Group: the smaller initial CDA amplitude averaged from loads 4 and 5, the more matches participants played [one unit decrease in the average CDA component’s amplitude resulted in an increase of 10.219 matches played (*t*_(39)_ = 2.077, *p* = 0.044); Figure 5A].

In the final analysis, we created a model containing a mean latency of first army unit creation as a predictor, Group as a moderator variable, and mean K obtained from post-training measurement as a dependent variable. Created model turned out to be significant [*F*_(3, 39)_ = 8.384, *p* < 0.001, *R^2^* = 0.392] and contained significant influence of the predictor [*b* = −0.009, *t*_(39)_ = −2.499, *p* = 0.017]. Group influence and interaction turned out to be insignificant (*p* = 0.349; *p* = 0.12; Figure 5B).

## Discussion

The study presented here examined the relationship between the RTS video game proficiency acquired during the training and the improvement of the VWM capacity indexed with behavioral and ERP measures. To properly inspect players’ game proficiency, telemetric data from the game environment were used. EEG and behavioral data were collected from non-gamers, who were assigned to one of three groups (Control Group, Fixed Group, and Variable Group).

Participants completed a change detection task, which is the typical experimental paradigm used to examine the VWM capacity, twice during their participation (pre-training and post-training in active groups or over a period of 4 weeks in the passive control group).

The obtained results suggest that VWM capacity improvement was the most significant in the group of participants with the Variable training model. This finding stands in agreement with our initial hypothesis, which assumes that video game influence may vary depending on the training model.

Most importantly, our results show that we can successfully explain game performance by looking at the initial values of the psychophysiological index of VWM and also the behavioral index of VWM (mean K value) at the post-training measurement can be predicted from in-game behavior.

We believe that natural predispositions are an important aspect of achieving success in training, but a good training environment is no less crucial. Therefore, potential players can reach their full potential only under the right conditions. The combination of aspects of natural predispositions and different training models allows for a better understanding of differences in the obtained results, but above all—it shows how important it is to control game environment conditions, which can diversify the gameplay in an enormous number of ways.

### VEG Participants Were Able to Achieve the Biggest Improvement of Their VWM Capacity During the Study

The participants from the group with the variable environment training model were able to significantly improve their VWM capacity (measured by Pashler’s formula of K value) on each of the tested loads (from load 2 to load 5). This after-training improvement in accuracy stands in agreement with studies, which show that AVG experience is related to VWM abilities (Green and Bavelier, 2003; Boot et al., 2008; Colzato et al., 2010; Clark et al., 2011; Blacker and Curby, 2013; Oei and Patterson, 2013; Li et al., 2015). Still, the Fixed Environment Group had only a significant increase on load 4 and load 5. Then it is important to emphasize that AVG influence corresponds to applied game mechanisms: SC2 matches require players to rapidly switch between multiple sources of action and information in general, but the training’s demands were different depending on the training’s model. A similar effect was not observed in the control group. Presented results argue that variable training strategies can be more beneficial and allow not only to achieve bigger improvement in specific task but also the occurrence of the far transfer. The fact that VEG players were able to achieve the biggest improvement of their VWM capacity after their training is consistent with this interpretation. In contrast, FEG players were not encouraged to thoroughly explore the game environment, learn different strategies and maximize their various skills, but rather, were trained to repeat one gameplay model in a non-engaging way.

### VEG Participants Were Able to Achieve the Biggest Game Proficiency

As mentioned above, three in-game indicators were chosen to measure game proficiency. (1) The number of played matches by each player; (2) latency of creating the first army unit; and (3) first supply collection latency. Telemetric data analysis shows us, even though there were no significant differences in groups about time spent on games, VEG players were able to play significantly more games in that period of time.

In comparison with FEG, VEG participants were significantly faster in creating their first army unit. However, there were no associations between the collection of first supply latency and group types.

These taken into account, we see that comparing with FEG, VEG settings allowed non-gamer participants to be greatly proficient in SC II.

### CDA Component, K Value, and Game-Related Factor Analysis

Neurophysiological output was closely analyzed with all parameters using repeated-measure ANOVA. Analyses did not pinpoint significant association either for group type or session. Yet, the load variable had a significant effect on mean CDA amplitudes. This means we observed different CDA amplitudes on different loads. Our data support the notion that CDA is a VWM indicator (Figure 4D).

Additionally, the K value had a correlation with CDA. Therefore we understand that low-valued CDA components are significantly associated with both increased VWM capacity and increased input on VWM.

### Game Proficiency Indicator Predicts VWM Capacity (K Value)

Two predictive models give us key insights about the relation between game performance, CDA, and K value obtained from the measurements. Model A holds a predictive value about the number of played SC II matches and the mean CDA amplitude on loads 4 and 5 (collected from pre-training session). Participants who have lower initial mean CDA amplitude are less likely to play a higher number of matches, which implies greater natural predispositions to succeed in the game environment. Then it needs to be highlighted that this model was only found to be significant for VEG. It shows that players’ natural predispositions can result in better in-game development only in a favorable environment.

Model B enables us to obtain information about participants’ level of VWM (K-value obtained from post-training measurement) just by looking at the latency of creating the first army unit. Such a model could help us (in the future) not only to create a rule of thumb for measuring VWM in a specific setting but also to determine players’ level of specific cognitive skills in a more natural environment.

Although performed analyzes did not reveal a significant model of moderated mediation, two independent regression models, it’s important to interpret obtained results in a broader, common context. As a complex game environment can be reflected by dozens of telemetric variables, which only together make up the full picture of the match and players’ skills, it may not be possible to create a simple and efficient model with only one telemetry variable.

Furthermore, initial VWN capacity, measured by K-value, didn’t determine in-game performance regardless of the analyzed indicator. Then behavioral results obtained from pre-training measurement cannot be clearly associated with participants’ natural predispositions. It should be noted then, that AVG requires more than one cognitive function, so the result of any single behavioral variable may turn out to be insufficient to fully reflect players’ in-game proficiency or predispositions.

Presented models, taken together, hold promising results for both: RTS gaming’s impact on VWM, and the role of neurophysiological indicators in recognizing the natural predispositions of AVG players. In conclusion, this study confirms that playing RTS games increases VWM capacity. As these improvements were majorly observed in VEG participants (yet still, FEG showed higher results in comparison with the control group), it can be assumed that the intensity of AVG influence depends on the adopted training model. What is more, in the presented study we propose a neurophysiological indicator, which may allow us to identify AVG players with higher predispositions to become better gamers. Last but not least: telemetric data sheds light on game performance, and combining it with other variables *via* regression models holds promising information as such, predicting the capacity of VWM (K-value, scored) from just one game proficiency indicator.

All these findings combined and experimental settings may hold a guiding reference for future research opportunities and commercial usage. Therefore it’s important to mention that future investigations should examine a wider range of carefully selected tasks, which can contribute to create a more complete spectrum of cognitive functions and changes that they undergo through VG training.

## Data Availability Statement

The raw data supporting the conclusions of this article will be made available by the authors, without undue reservation.

## Ethics Statement

The studies involving human participants were reviewed and approved by Komisja ds. Etyki Badań Naukowych Wydziału Psychologii w Warszawie [Ethics committee of Department of Psychology at University of Social Sciences and Humanities]. The patients/participants provided their written informed consent to participate in this study.

## Author Contributions

NJ: analyzed data, prepared figures, and wrote the first version of the manuscript. PD: designed the study and prepared paradigms’ code. IA: helped with EEG data preparation and analysis. MM: collected data. ABr: study conceptualization, data interpretation, manuscript correction, and final approval. All authors contributed to the article and approved the submitted version.

## Conflict of Interest

The authors declare that the research was conducted in the absence of any commercial or financial relationships that could be construed as a potential conflict of interest.

## Publisher’s Note

All claims expressed in this article are solely those of the authors and do not necessarily represent those of their affiliated organizations, or those of the publisher, the editors and the reviewers. Any product that may be evaluated in this article, or claim that may be made by its manufacturer, is not guaranteed or endorsed by the publisher.
